# Metal-organic frameworks enable broad strategies for lithium-sulfur batteries

**DOI:** 10.1093/nsr/nwab055

**Published:** 2021-04-15

**Authors:** Cheng Zhou, Zhaohuai Li, Xu Xu, Liqiang Mai

**Affiliations:** State Key Laboratory of Advanced Technology for Materials Synthesis and Processing, Wuhan University of Technology, Wuhan 430070, China; State Key Laboratory of Advanced Technology for Materials Synthesis and Processing, Wuhan University of Technology, Wuhan 430070, China; State Key Laboratory of Advanced Technology for Materials Synthesis and Processing, Wuhan University of Technology, Wuhan 430070, China; International School of Materials Science and Engineering, Wuhan University of Technology, Wuhan 430070, China; State Key Laboratory of Advanced Technology for Materials Synthesis and Processing, Wuhan University of Technology, Wuhan 430070, China; Foshan Xianhu Laboratory of the Advanced Energy Science and Technology Guangdong Laboratory, Foshan 528200, China

**Keywords:** metal-organic frameworks, lithium-sulfur batteries, cathode, separator, electrolyte

## Abstract

The lithium-sulfur (Li-S) battery is considered to be a potential next-generation power battery system, however, it is urgent that suitable materials are found in order to solve a series of challenges, such as the shuttle effect and lithium dendrite growth. As a multifunctional porous material, metal-organic frameworks (MOFs) can be used in different parts of Li-S batteries. In recent years, the application of MOFs in Li-S batteries has been developed rapidly. This review summarizes the milestone works and recent advances of MOFs in various aspects of Li-S batteries, including cathode, separator and electrolyte. The factors affecting the performance of MOFs and the working mechanisms of MOFs in these different parts are also discussed in detail. Finally, the opportunities and challenges for the application of MOFs in Li-S batteries are proposed. We also put forward feasible solutions to related problems. This review will provide better guidance for the rational design of novel MOF-based materials for Li-S batteries.

## Teaser

This review summarizes the recent advances of metal-organic frameworks in lithium sulfur batteries, providing feasible solutions to overcome the challenges and the guidance for the further development.

## INTRODUCTION

With the rapid increase in demand for electric vehicles and mobile devices, the demand for new energy storage devices is increasing [1]. The rapid development of the electric vehicle industry has put forward higher requirements for the safety, energy density, cycle life and cost of power batteries [2,3]. At present, the energy density of commercial lithium-ion (Li^+^) batteries is difficult to break through 300 Wh kg^–1^. Therefore, it is imperative to accelerate the development of high-energy-density battery systems based on new reaction mechanisms [4–6]. The theoretical capacity of elemental sulfur is as high as 1675 mAh g^–1^, which possesses the advantages of abundant reserves, low price and environmental friendliness. Therefore, it is considered to be one of the most promising of the potential next-generation cathode materials [7]. When lithium metal with a theoretical capacity of 3860 mAh g^–1^ is used as the anode to match with the sulfur cathode, the theoretical energy density of the battery can reach 2600 Wh kg^–1^. Therefore, the lithium-sulfur (Li-S) battery system is considered to be one of the emerging energy storage systems that is expected to break through 500 Wh kg^–1^ at the cell level [8,9]. For a

long time, it has been recognized that the problems of the sulfur cathode are the main factor restricting the development of Li-S batteries, including the low conductivity of sulfur, volume expansion and shuttle effect. After years of research, the above problems have been widely solved through the design of the cathode or separator, and the capacity and cycle life of Li-S batteries has been significantly improved [10,11]. However, in recent years, it has been found that lithium dendrite growth, volume expansion and an unstable solid electrolyte interface (SEI) of the lithium metal anode also seriously affect the performance of Li-S batteries [12,13]. More importantly, when the battery is assembled into pouch cells for testing, some problems of the lithium metal anode become more prominent, which seriously affects the cycle life of the Li-S battery system and induces potential safety hazards [14]. Similarly, in order to solve the problems of the lithium metal anode, stable lithium/electrolyte interfaces and a complex lithium anode structure are mainly designed to improve its stability, including the preparation of an artificial SEI or interface layer, optimization of electrolyte composition and additives, and construction of lithium deposition frameworks.

**Figure 1.**
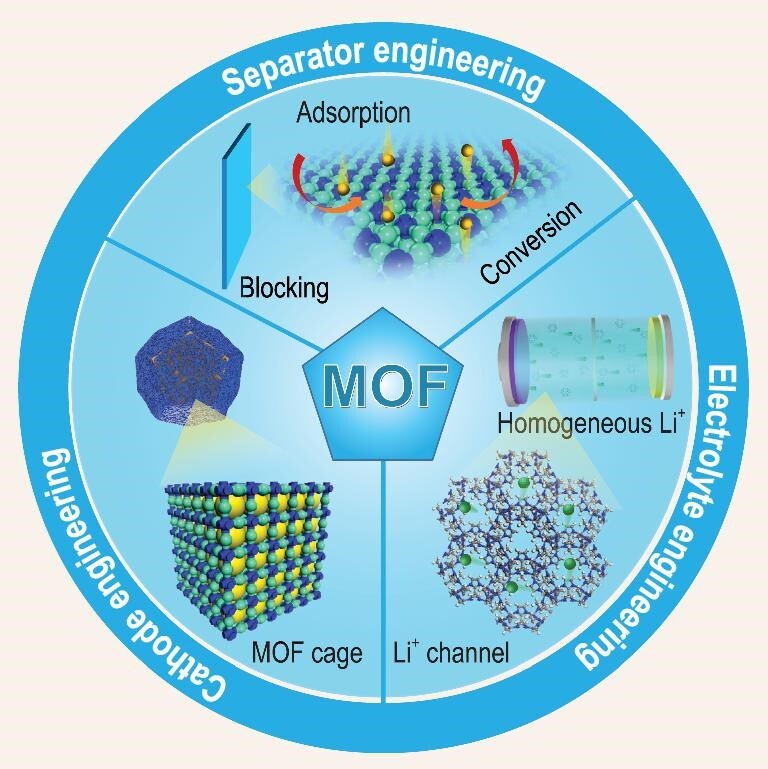
Schematic illustration of the working mechanisms of MOFs in different parts of Li-S batteries.

Researchers are committed to finding suitable materials to improve the performance of Li-S batteries. A large number of different materials have been used in the modification of the sulfur cathode, separator and electrolyte [15,16]. Among them, known as porous coordination polymers, metal organic frameworks (MOFs) stand out due to their intrinsic features. Different from the traditional porous materials, MOFs, which are composed of metal ions and organic ligands, possess a highly ordered structure, tunable pore size and a hybrid inorganic–organic nature. The different characteristics of MOFs can be applied when solving different problems of Li-S batteries (Fig. 1). For example, the tunable pore size and highly ordered structure of MOFs enables them to be used as sulfur hosts [17]. At the same time, the central metal ions and organic ligands can effectively adsorb polysulfides and act as catalytic sites to accelerate the transformation of polysulfides (Fig. 1). In a word, traditional porous materials, such as porous carbon materials, generally do not have polarity, and only have physical limitations for polysulfides, while other polar materials, such as transition metal oxides, generally do not possess the appropriate pore structure, making it difficult to achieve sulfur loading, and they must be combined with other materials. Significantly, MOFs possess both porous and polar characteristics, which can not only achieve uniform sulfur loading but also provide physical limitation and chemical adsorption for polysulfides. However, most of the MOFs are non-conductive, which limits their application in the sulfur cathode to some extent.

Therefore, some studies suggest that MOFs are more suitable as the coating layer of the separator [18]. In addition to the above characteristics, compared with traditional carbon materials, the insulating character of MOFs make the polysulfides difficult to deposit on, which thus prevents subsequent retrace of polysulfides to the sulfur cathode due to the concentration gradient. In the meantime, its one-dimensional (1D) pore can serve as a diffusion channel for Li^+^, which can effectively homogenize the deposition of Li^+^ and protect the lithium metal anode (Fig. 1). This ion sieving effect of MOFs can also be applied to the design and construction of artificial SEI films for lithium metal anodes and as an additive for electrolytes. More recently, as an excellent ion sieve with mesoscopic channels, MOFs have also attracted great interest in the area of solid-state electrolytes (SSEs) based on single ion conductors [19], which can significantly inhibit the growth of lithium dendrites in the lithium metal anode.

In conclusion, MOFs are a versatile material for Li-S batteries and can be used for the improvement of various components of Li-S batteries. However, the current review on the application of MOFs in Li-S batteries puts the attention on the MOF derivatives, or focuses on only one part of the Li-S battery [20,21]. Therefore, the important characteristics of pristine MOFs, beneficial for Li-S batteries, are ignored. In this review, we emphasize the ground-breaking works and recent advances of pristine MOFs for Li-S batteries. The main text is developed according to the roles of MOFs in Li-S batteries and divided into three parts: (i) MOF-based composite sulfur cathode, (ii) MOF-based functional separator, and (iii) MOF-based electrolyte. Through the detailed discussion of the pioneering and representative works of MOFs in Li-S batteries, the working mechanisms of MOFs in different components of the Li-S battery are analyzed and revealed. Furthermore, combined with the current research progress, the existing problems of MOFs in Li-S batteries are summarized, and the possible solutions are also mentioned. In the end, the potential direction and prospects of the application of MOFs in Li-S batteries are proposed.

## MOF-BASED COMPOSITE SULFUR CATHODE

The reaction mechanism of the Li-S battery is different from that of the traditional Li^+^ battery. The Li-S battery is based on a multi-step redox reaction that creates electron migration during the discharge process. The sulfur cathode will expand up to 80% in volume [22] and a series of intermediate polysulfides will be produced. The main reactions are as follows:

(1) \begin{equation*}{{\rm{S}}_{{\rm{(s)}}}} \leftrightarrow {{\rm{S}}_{{\rm{(l)}}}},\end{equation*}

(2) \begin{equation*}{{\rm{S}}_{\rm{8}}}{\rm{\ + \ 2}}{{\rm{e}}^{\rm{ - }}} \leftrightarrow {\rm{S}}_{\rm{8}}^{{\rm{2 - }}},\end{equation*}

(3) \begin{equation*}{\rm{3S}}_{\rm{8}}^{{\rm{2 - }}}{\rm{\ +\ 2}}{{\rm{e}}^{\rm{ - }}} \leftrightarrow {\rm{4S}}_{\rm{6}}^{{\rm{2 - }}},\end{equation*}

(4) \begin{equation*}{\rm{2S}}_{\rm{6}}^{{\rm{2 - }}}{\rm{\ +\ 2}}{{\rm{e}}^{\rm{ - }}} \leftrightarrow {\rm{3S}}_{\rm{4}}^{{\rm{2 - }}},\end{equation*}

(5) \begin{equation*}{\rm{S}}_{\rm{4}}^{{\rm{2 - }}}{\rm{\ +\ 2}}{{\rm{e}}^{\rm{ - }}} \leftrightarrow {\rm{2S}}_{\rm{2}}^{{\rm{2 - }}},\end{equation*}

(6) \begin{equation*}{\rm{S}}_{\rm{2}}^{{\rm{2 - }}}{\rm{\ + \ 2}}{{\rm{e}}^{\rm{ - }}} \leftrightarrow {\rm{2}}{{\rm{S}}^{{\rm{2 - }}}},\end{equation*}

(7) \begin{equation*}{\rm{S}}_{\rm{2}}^{{\rm{2 - }}}{\rm{\ +\ 2L}}{{\rm{i}}^{\rm{ + }}} \leftrightarrow {\rm{L}}{{\rm{i}}_{\rm{2}}}{{\rm{S}}_{\rm{2}}}\! \downarrow, \end{equation*}

(8) \begin{equation*}{{\rm{S}}^{{\rm{2 - }}}}{\rm{ \ +\ 2L}}{{\rm{i}}^{\rm{ + }}} \leftrightarrow {\rm{L}}{{\rm{i}}_{\rm{2}}}{\rm{S}}\! \downarrow \!. \end{equation*}

In the discharge process, the solid sulfur is dissolved in the electrolyte firstly, then gradually reduced to soluble long-chain polysulfides (Li_2_S_x_, x = 4–8), and finally transformed into insoluble Li_2_S_2_ and Li_2_S. Since the electrolyte used in Li-S batteries is ether-based, the long-chain polysulfides (Li_2_S_x_, x = 4–8) produced during the cycle will dissolve in the electrolyte [23]. Due to the effect of concentration difference, soluble polysulfides will pass through the separator and directly reach the lithium metal anode. Furthermore, due to the effect of the electric field, some polysulfides will return to the cathode again, so as to form a ‘shuttle effect’. The shuttle effect will seriously affect the ion migration rate, causing delayed battery dynamic reaction, which will reduce the utilization rate of sulfur and eventually lead to the fast attenuation of capacity and Coulombic efficiency. Commercial conductive carbon materials, such as acetylene black and Ketjen black, have weak binding force with polysulfides and cannot effectively trap polysulfides. Therefore, it is necessary to find suitable materials to accommodate the volume change of the sulfur cathode and inhibit the shuttle effect of the Li-S battery. Due to its regular structure, large specific surface area and uniform but controllable pore structure, the MOF is considered to be one of the preferred porous materials for a sulfur cathode host [24]. Compared with traditional porous materials, the metal atom centers (Lewis acidic sites) and organic ligands (Lewis basic sites) of MOFs can provide more active sites, which can effectively adsorb polysulfides and confine them inside MOFs. In particular, the porous nanocomposites with abundant cage structures provide the possibility for researchers to design novel materials at the molecular level that can effectively inhibit the dissolution and diffusion of polysulfides in the electrolyte. Therefore, a large number of studies have focused on the application of pristine MOFs and MOF composites in the cathode host of Li-S batteries. Related works are summarized and discussed in detail in this section.

### Pure MOFs

The milestone work of MOFs in Li-S batteries can be traced back to 2011. Tarascon *et al.* proposed a mesoporous chromium trimesate MOF named MIL-100(Cr) as the host material for sulfur impregnation [17]. Owing to its large pore volume and unique mesoporous structure, it is suitable for sulfur loading by melting diffusion method. The pores distributed on the surface can also contain a certain amount of electrolyte to enhance the ionic conductivity of the electrode. It is also pointed out that, although the insulation of the MOF seems to affect its use as the host material of sulfur based on the reaction mechanism of the Li-S battery, the elemental sulfur will be converted into soluble polysulfide after the first discharge process, and will never return to the elemental sulfur in the next cycle. So regardless of the initial sulfur powder, the reaction of the sulfur cathode can be regarded as the reaction of the electrolyte. Therefore, the pore structure is more important than the conductivity. This work has greatly promoted the application of pure MOFs in the cathodes of Li-S batteries. Since then, exciting progress has been made in the application and optimization of various MOFs in Li-S batteries.

**Figure 2.**
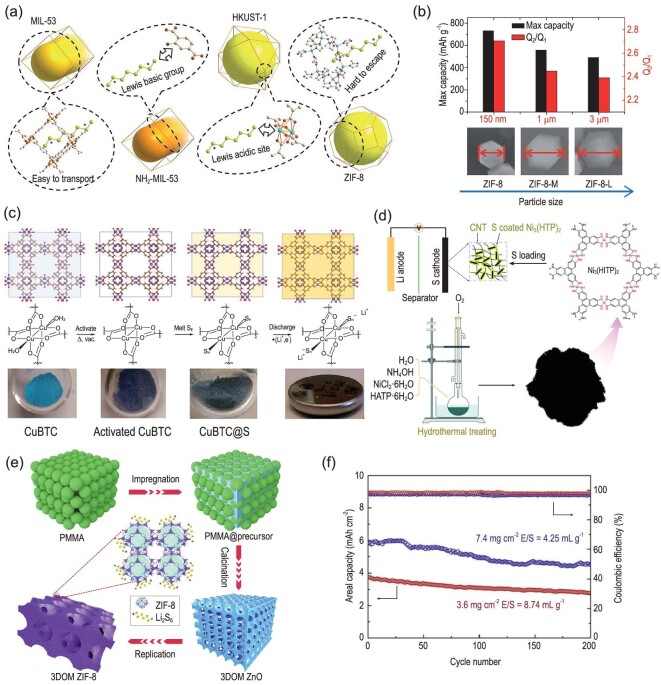
(a) Schematic of the MOFs chosen in this study and their unique characteristics. (b) The dependence of maximum discharge capacity and Q2/Q1 on the particle size. Adapted with permission from [25]. Copyright 2014, Royal Society of Chemistry. (c) Activation and sulfur loading of CuBTC. Adapted with permission from [26]. Copyright 2018, Royal Society of Chemistry. (d) Schematic diagram of the synthesis of a Ni_3_(HITP)_2_/S cathode. Adapted with permission from [29]. Copyright 2019, Wiley-VCH. (e) Schematic illustration of the 3DOM ZIF-8 synthesis process. (f) Cycling performance at 0.2 C of S/3DOM ZIF-8 electrodes under high sulfur loading and limited electrolyte. Adapted with permission from [30]. Copyright 2020, Elsevier Ltd.

However, in the early stage, as to the application of MOFs in the sulfur host, there were some problems, such as fast initial capacity attenuation, low areal sulfur loading and poor rate performance. Based on the previous experience of intercalation reaction cathode, Zhou *et al.* concluded that the size of MOFs matters a lot as sulfur host material [25]. Four MOFs with distinct structures were selected as sulfur hosts to verify this point (Fig. 2a). Through comparative analysis of the electrochemical performance test results, it can be seen that all MOF/S composite cathodes have an activation process in the initial stage, which is consistent with the early work. The activation time of MOFs with small pore size is much longer. After the activation process, the capacity of the three MOFs with small size is more than 1000 mAh g^–1^ and ZIF-8 with the smallest crystals size shows better rate performance, while the capacity of HKUST-1 with the size of ∼10 μm is only 526 mAh g^–1^. This is precisely the larger size that seriously affects the migration rate of Li^+^. In order to further study how the particle size affects the performance of the MOF-based composite sulfur cathode, three kinds of ZIF-8 particles with different sizes (150 nm, 1 μm, 3 μm) were prepared. By comparing the cycling performances of sulfur cathodes based on different MOFs at a current density of 0.5 C, it can be seen that ZIF-8 with an average size of 150 nm can achieve higher maximum capacity (Fig. 2b). The results indicate that the reduction of MOF particle size can effectively shorten the diffusion lengths, accelerating the Li^+^ migration rate and improving the utilization rate of elemental sulfur. Therefore, after determining the types of MOFs, we can further tailor the particle size to choose a more ideal MOF host, so as to obtain a composite sulfur cathode with better performance.

The factors influencing the performance of MOF-based sulfur cathodes were also analyzed in some works, but different mechanisms were explained. Baumann *et al.* selected CuBTC (HKUST-1) as the research target, and analyzed the influence of MOF structure and chemical properties on polysulfide adsorption and battery performance [26]. It was suggested that the central metal sites play an important role in the performance of MOF-based hosts (Fig. 2c). Firstly, it was proved by theoretical calculation that the Cu Lewis acidic centers are unsaturated and can accept the electron density of S clusters, thus forming strong Cu-S interactions. Furthermore, the existence of Cu-S interactions was proved by X-ray absorption spectroscopy and other characterization methods. The results showed that the numbers of Cu-S interactions increased with the increase of sulfur loading. However, the EXAFS data analysis showed that when the sulfur content increases to a certain extent, the increase of Cu-S interactions is not obvious. This non-linear relationship is mainly caused by the low efficiency of S migration to Cu sites. When the sulfur loading is high, it is very difficult for S clusters to diffuse to the central Cu site through the channels on the MOF surface. It was also found that the smaller MOF particles contain higher density Cu sites, so more Cu-S can be formed. Therefore, the authors also believed that small-sized MOFs can adsorb polysulfides more effectively, which is consistent with the research results of earlier work. However, it is different from previous work that showed that small-sized MOF hosts possess higher Li^+^ migration rate. The author put forward a new viewpoint to explain the reason for the improvement of cathode performance from the perspective of metal active sites. This work is a supplement to the early work, and provides a more sufficient basis for the selection of appropriate MOF hosts for Li-S batteries. As we know, MOFs rely on the pores on the surface to achieve sulfur loading, and the same MOF has the same pore structure, so the adaptabilities of the same MOF with different sizes to sulfur are similar. In the case of the same mass, the small-sized MOF possesses higher specific surface area, so it can achieve higher sulfur loading. At the same time, based on the existing works, the small-sized MOF possesses higher Li^+^ migration rate and higher metal site density than the large-sized MOF, and can still maintain good reaction kinetics and effective polysulfide adsorption when sulfur loading is high. Therefore, the small-sized MOF is more conducive to a stable cycle under high sulfur loading. Based on the size effect of MOFs on the performance of the cathode, Hong *et al.* also synthesized a bi-functional Cu MOF (Cu-TDPAT) based on the microwave method [27]. Cu-TDPAT is formed by the coordination of Cu^2+^ with organic ligand 2,4,6-tris(3,5-dicarboxylphenylamino)-1,3,5-triazine (H_6_TDPAT), which contains abundant nitrogen functional groups. Therefore, in this structure, Cu open metal sites can combine with polysulfide anions, and the nitrogen-containing functional sites on the ligand can coordinate with lithium ions. The synergistic effect of the ligands and Cu open metal sites can make it adsorb polysulfides effectively.

With the development of the application of MOFs in Li-S batteries, the above factors affecting the selection of MOFs for sulfur hosts are well known, and some works began to study MOFs with special properties or to design the desirable structure for the sulfur host [28]. A highly conductive MOF named Ni_3_(HITP)_2_ was used as the cathode host of Li-S batteries for the first time by Cai *et al.* (Fig. 2d) [29]. Due to its strong π–π conjugate structure and weak metal–metal interaction, the test results show that the conductivity of Ni_3_(HITP)_2_ at room temperature is as high as 200 S m^–1^. At the same time, the influence of conductivity on the kinetics of the Li-S battery was analyzed by multi-scanning rates CV test. The test results show that the Ni_3_(HITP)_2_/S composite cathode possesses higher Li^+^ diffusion coefficients than other composites in different cycling stages, which can accelerate the transition of intermediate polysulfides and improve the dynamics of the Li-S battery. As a result, the sulfur utilization rate, cycle performance and rate performance of the Li-S battery with Ni_3_(HITP)_2_/S composite cathode has been significantly improved. Cui *et al.* constructed three dimensionally ordered macro microporous (3DOM) MOFs based on the template method (Fig. 2e) [30]. The pre-synthesized poly(methyl methacrylate) (PMMA) nanospheres with uniform size were immersed in Zn(NO_3_)_2_•6H_2_O solution with citric acid as the chelating agent, and then calcined to form a 3D porous ZnO framework. Finally, after the complete coordination of Zn^2+^ with 2-methylimidazole, 3D ZIF-8 was prepared as the sulfur host. Uniform sulfur loading and good electrolyte infiltration can be achieved with the 3DOM framework, and the microporous ZIF-8 nanoparticles can effectively adsorb the polysulfides. Due to the synergistic effect of this structure, the shuttle effect was effectively suppressed and sulfur reaction kinetics were promoted. The higher Li^+^ diffusion coefficient of this structure is also proved by CV curves with different scan rates. As for the electrochemical performance, the capacity of the S/3DOM ZIF-8 cathode can be maintained at 674 mAh g^–1^ after 500 cycles at 2 C, and the decay rate is only 0.028% per cycle. Even when the areal sulfur loading is as high as 7 mg cm^–2^ and only 4.25 ml g^–1^ of the electrolyte is used, a high initial areal capacity of 5.8 mAh cm^–2^ can be achieved, and 4.5 mAh cm^–2^ can be maintained after 200 cycles, which further proves the advantages of this novel structure (Fig. 2f).

In another work, Li *et al.* analyzed the possibility of three transition metal hexaminobenzene-based coordination polymers (TM-HAB-CPs) for the cathodes of Li-S batteries [31]. The results of DFT calculation show that vdW interaction and solvent effect must be considered when calculating the adsorption energy of polysulfides on the new 2D MOFs. Based on a series of calculation results, it is concluded that V-HAB-CPs have the largest adsorption energy for polysulfides, and its structure is most stable in the electrochemical reaction process. Based on the sandwich structure, the volume change of V-HAB-CPs is only 3.06%. Compared with Cr and Fe-HAB-CPs, V-HAB-CPs are more likely to be used as the sulfur host of Li-S batteries. The proposed calculation method and model can also be used to calculate other materials in Li-S batteries. More recently, a MOF based on Fe (III) trimers called MIL88-A was employed as the sulfur host for an Li-S battery by Benítez *et al.* [32]. MIL88-A is a kind of semiconductor material, which can effectively promote the electron transport.

### MOF composites

**Figure 3.**
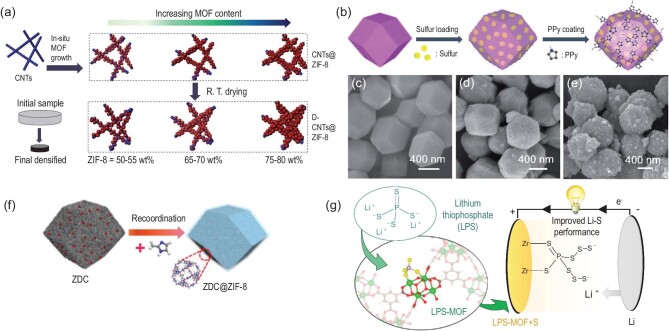
(a) Schematic illustration of the fabrication process including *in situ* MOF growth and R. T. drying. Adapted with permission from [36]. Copyright 2019, Royal Society of Chemistry. (b) Schematic illustration of the preparation process of ZIF-67-S-PPy. (c–e) SEM images of ZIF-67 nanocrystals, ZIF-67-S-60% and ZIF-67-S-PPy-60%, respectively. Adapted with permission from [43]. Copyright 2019, Royal Society of Chemistry. (f) Schematic of the fabrication of ZDC@ZIF-8. Adapted with permission from [44]. Copyright 2019, Elsevier Ltd. (g) Schematic illustration of lithium thiophosphate functionalized zirconium MOFs for Li-S batteries. Adapted with permission from [45]. Copyright 2019, American Chemical Society.

Although MOF hosts have improved the performance of Li-S batteries, due to the limitations of the insulation characteristics and structure of MOFs, the rate capability and sulfur content of MOF-based hosts are generally low. Therefore, some researchers began to combine MOFs with other materials to solve the above problems while giving full play to the advantages of MOFs. Carbon nanotubes (CNTs) are widely used for sulfur loading due to their high specific surface area and excellent electrical conductivity [33]. Xu *et al.* combined MOFs and CNTs by a one-pot method [34]. The Ni-MOF-74/CNT composite was successfully prepared by *in situ* modification of the MOF on CNT. The effective polysulfide adsorption of Ni-MOF-74 and good conductivity of the CNT make the composite sulfur cathode show excellent rate performance and cycle performance. Zhang *et al.* also prepared a ZIF-8 modified self-supporting composite sulfur cathode based on 3D CNT foam [35]. On the basis of this work, more recently Zhang *et al.* proposed that there is a large gap between MOF particles, which means the larger area cannot be effectively used. Therefore, the authors densified the composite structure by a simple drying-shrinkage process at room temperature (Fig. 3a) [36]. In this structure, CNTs are used as a conductive framework for ion transport, and the MOF particles contact with each other to fill the gap. By adjusting the size and content of MOFs, a dense 3D network structure with high specific area and high porosity is realized, which can achieve higher sulfur content. Therefore, compared with the conventional MOF/CNT composite cathode, higher area and volume specific capacity has been achieved by this novel structure. A thiol-modified CNT@UIO66 composite structure was used as the host by Liu *et al.* [37]. In this structure, S and UIO-66-SH can be covalently linked to prevent the dissolution of polysulfides. As a result, the CNT@UIO-66/S cathode shows a good cycle stability. The capacity decay rate is only 0.017% per cycle after 450 cycles at 1 C. Even after 900 cycles at 2 C, the capacity retention rate is as high as 80.19%. This work also introduces a new concept for the role of MOFs in the cathodes of Li-S batteries. In addition to CNT, graphene [38] and reduced graphene oxide (rGO) [39,40] are also used to combine with MOFs to improve the performance of sulfur cathodes.

Conductive polymer coating is another effective strategy to improve the conductivity of MOF-based hosts [41,42]. Geng *et al.* firstly used ZIF-67 for sulfur loading, and then a layer of PPy was uniformly coated on the surface of ZIF-67/S composite through subsequent liquid-phase reaction [43], thus forming a novel hollow structure (Fig. 3b–e). The inner hollow structure can effectively withstand the volume expansion of the sulfur cathode, while the PPy layer coated on the surface can improve the conductivity of the cathode. Contrary to the above strategy, our group designed a composite structure with MOF to coat porous carbon [44]. Based on the low-pressure chemical vapor deposition (LPCVD) method, 2-methylimidazole vapor was re-coordinated with Zn^2+^ on the surface of the porous carbon, and a thin layer of ZIF-8 was uniformly modified on the surface of the porous carbon, thus forming a core-shell structure (Fig. 3f). Compared with previous works, in this novel structure, a higher sulfur content can be realized when the porous carbon is used as the inner hollow structure. The outer ZIF-8 layer can effectively inhibit the dissolution of polysulfide without increasing the cathode loading.

More recently, Baumann *et al.* found that MOFs can be functionalized by pre-coordination with other substances, which can effectively improve sulfur utilization and polysulfide encapsulation [45]. Li_3_PS_4_ was used as guest molecules to bind with the open sites in Zr-MOFs (Fig. 3g**)**. It is also proved by a series of characterization methods that the functionalized MOF structure is very stable due to the combination of thiophosphate and Zr open sites. The PS_4_^3–^ group can form a reversible S−S bond with polysulfides generated during the cycle, effectively preventing the dissolution of polysulfides and phosphates into the electrolyte. Another work about lithiated MOFs with a similar mechanism was also done by Baumann and co-workers [46].

In brief, this part highlights the application of MOFs and MOF composites for the cathode of Li-S batteries. The mechanisms of MOFs in the cathode of Li-S batteries, and the factors influencing and methods to improve the performance of MOF-based cathodes are summarized. However, although MOFs have some advantages over traditional porous materials, the application of most MOFs in the sulfur cathode is limited by their insulation properties. Therefore, MOF composites can make up for the defects of MOFs and make full use of the advantages of MOFs. At the same time, it is highly justified to develop novel conductive MOFs or functionalized MOFs as sulfur hosts for Li-S batteries. On the other hand, considering the commercial application, the synthesis conditions and production cost of MOFs are also problems to be considered when selecting appropriate MOF hosts. At present, the performance test of Li-S batteries is usually based on coin cell, in which Li is unlimited and the electrolyte is largely excessive. When considering the commercial development of high energy density Li-S batteries, the negative/positive capacity (N/P) ratio must be considered. However, due to the existence of the shuttle effect and side reaction, lithium and sulfur will be continuously consumed during the cycling, resulting in the mismatch of positive and negative electrode capacity, especially with a high sulfur loading cathode. Therefore, in order to obtain the balanced Li-S battery, the key is to solve the shuttle effect and realize the formation of stable SEI film on the surface of the lithium anode.

## MOF-BASED FUNCTIONAL SEPARATOR

Although the volume expansion of sulfur can be effectively alleviated and the dissolution of soluble polysulfide can be restrained by applying a host for the sulfur cathode, the active material content of the cathode will be inevitably reduced. The separator is an important part of the battery, located between the cathode and anode for effective ion transport and electron block to prevent short circuit. Due to the existence of soluble polysulfides during the cycling, the requirements for the separator of Li-S batteries are higher than that of Li^+^ batteries. At present, commercial polyolefin separator is widely used for Li-S batteries. In order to achieve good Li^+^ transport, this type of separator contains a large number of nano-sized pores. However, this structure also means the soluble polysulfides are easily transported to the anode through the membrane, and directly react with lithium metal, causing a series of side reactions. In order to solve the problems of polyolefin separator, research mainly focuses on the functional modification of polyolefin separators or the construction of an interlayer between the cathode and the separator. Recently, some novel functional separators for Li-S batteries have also been designed [47,48]. MOFs can not only realize the effective adsorption of polysulfides and act as a catalytic center to promote the transformation of polysulfides, but also can work as an ion sieve for the separator because of their ion selectivity. Therefore, some important works and the recent advances of MOFs with regard to separator modification and novel separators of Li-S batteries are summarized in this part.

### MOF-particle-modified separator

**Figure 4.**
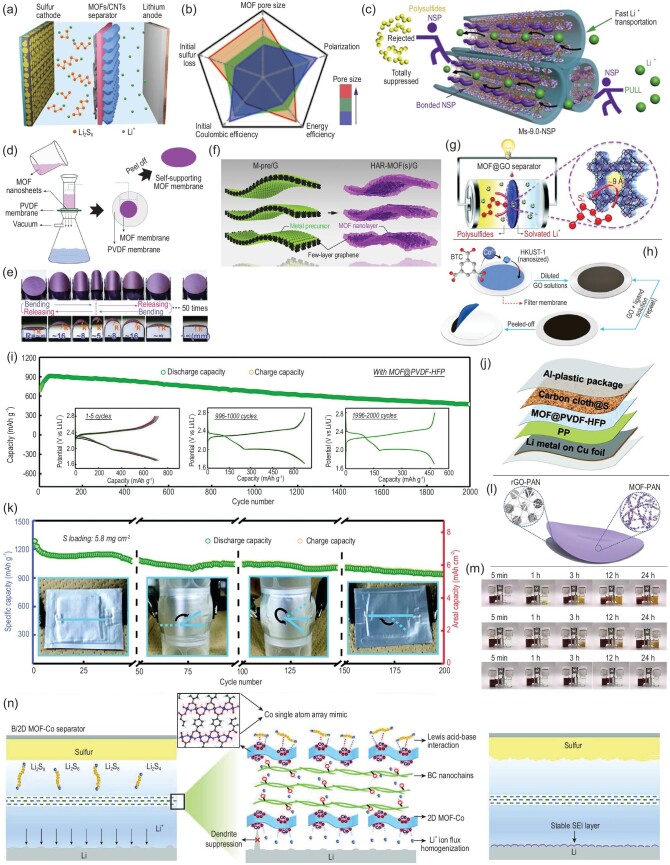
(a) Schematic for Li-S battery MOF/CNT-modified separator. Adapted with permission from [52]. Copyright 2017, American Chemical Society. (b) The effect of pores on the performance of Li-S batteries. (c) Schematic mechanism of the Ms-9.0-NSP. Adapted with permission from [53]. Copyright 2020, Elsevier Ltd. (d) Schematic image for the preparation of a Cu_2_(CuTCPP) membrane. (e) Bending and relaxing processes of the membrane. Adapted with permission from [65]. Copyright 2019, Elsevier Ltd. (f) Schematic illustration of MOF/graphene nanosheet preparation. Adapted with permission from [66]. Copyright 2020, American Chemical Society. Schematic of (g) MOF@GO separators in Li-S batteries and (h) the fabrication process. Adapted with permission from [71]. Copyright 2016, Nature Publishing Group. (i) Ultralong-term cycling performance of an Li-S cell. Schematic of (j) the inner structure and (k) cycling performance of the flexible Li-S pouch cell with an MOF@PVDF-HFP separator. Adapted with permission from [72]. Copyright 2018, Wiley-VCH. (l) Schematic of the MOF-PAN/rGO-PAN separator. (m) Permeation experiments for different separators. Adapted with permission from [74]. Copyright 2020, Elsevier Ltd. (n) Schematic illustration for the Li-S batteries with Celgard and B/2D MOF-Co separators. Adapted with permission from [76]. Copyright 2020, Wiley-VCH.

Similar to the cathode, MOF particles were first used to modify the polyolefin separator [49–51]. For a long time, the factors affecting the inhibition effect of MOF-modified separators on the shuttle effect were not discussed in detail, and the working mechanism of MOF-based separators has not been fully revealed. Therefore, some work has been devoted to the analysis of factors affecting the performance of MOF-based separators. Li *et al.* compared the inhibition effect of various MOFs (with different pore size and chemical structures) on the shuttle effect [52]. Four kinds of MOFs, including Y-FTZB, ZIF-7, ZIF-8 and HKUST-1, were used to modify the separators of Li-S batteries (Fig. 4a). As we know, the cycling performance is the best response to the capability of the separator to mitigate the polysulfide diffusion. The electrochemical test showed that the separator modified by Y-FTZB possesses the highest initial capacity and the best cycle stability, followed by ZIF-7-, ZIF-8-, CNT- and HKUST-1-based separators. However, this result does not follow the sequence of pore size, and ZIF-7 with the smallest pore size does not show the best performance, which means that the pore size is not the only factor affecting the performance of MOFs. Based on the results of SEM imaging, it is considered that the packing density of MOF particles is the key factor affecting the performance of separators modified by MOFs. The modified layer with a dense structure shows better cycling performance. However, the effect of pore size of the MOF on the performance of modified separators for Li-S batteries is not discussed in detail.

Recently, Chang *et al.* studied the effect of pore size and metal sites on the performance of MOF-based separators [53]. Through the electrochemical performance test and Raman spectrum analysis, it is found that the interaction between polysulfides and metal sites in MOF pores will produce a lot of ‘dead sulfur’, resulting in capacity loss and Coulombic efficiency reduction. This phenomenon is more obvious for MOFs with large pore size. By reducing the pore size of MOFs, the initial loss of sulfur can be reduced effectively, but the energy barrier of Li^+^ migration is also increased, which aggravates the polarization effect of the battery. The results verified that the MOF properties indeed affect the electrochemical performance of Li-S batteries (Fig. 4b). Based on this rule, a negatively charged sulfonic polymer (NSP) was used to modify the channels of CuBTC (with a pore size of 8.2 Å) (Fig. 4c), so as to reduce the pore size and eliminate the metal–sulfur interaction. The positive effect of NSP was further confirmed by the experimental results. Compared with the unmodified MOF, the modified MOF exhibited lower polarization voltage, higher initial capacity and lower capacity attenuation, which indicated that the introduction of NSP can effectively reduce the initial sulfur loss and the Li^+^ migration barrier. This work not only revealed the significant role of pore size and metal sites in MOFs, but also proposes a new strategy to improve the properties of MOFs, which can provide significant guidance for designing MOF-based separators for Li-S batteries. However, MOFs modified on the separator did not form a continuous and complete layer, and the polysulfides could still pass through the separator through the gaps between MOF particles. Therefore, the conclusions obtained in the above two works must be taken into account when choosing the appropriate MOF for modifying the separator of an Li-S battery. In addition to the MOFs mentioned above, more recently, Ti-containing [54] and Zr-based [55] MOF practices have also been used to modify the Li-S battery separator.

Since it is difficult for MOF particles to form a continuous modification layer, some studies have combined the MOF with other substances to form composites as coating materials for Li-S batteries [56–60]. Recently, inspired by the fact that CeO_2_ can effectively adsorb and catalyze polysulfides, Hong *et al.* prepared Ce-MOFs/CNT composites for separator coating [61]. The effective adsorption and catalytic effect of Ce-MOF on polysulfides was proved by a series of characterizations. The Ce-MOF/CNT-modified separators also showed good cycle performance and rate performance.

### 2D MOF-modified separator

Early experience tells us that 2D materials have advantages when they are used to construct thin films because of their larger surface/volume ratio than bulk materials [62]. Therefore, 2D MOFs are also used to prepare modified layers to block polysulfides. A conductive MOF with a 2D layered structure was used for separator modification by Zang *et al.* [63]. Based on a simple liquid-solid reaction, a continuous crack-free conductive Ni_3_(HITP)_2_ film can be formed *in situ* on the separator. Due to the uniform 1D channels and high conductivity, the rate performance and cycle performance of Li-S batteries assembled with the modified separator have been significantly improved. The advantages of the modified layer were further proved by using a S/CNTs cathode with high areal sulfur loading of 8.0 mg cm^–2^. After 200 cycles at 0.5 C, a high areal capacity of 7.24 mAh cm^–2^ can still be achieved. It is worth noting that the thickness of the Ni_3_(HITP)_2_-modified layer is only 340 nm, and the areal mass loading is only 0.066 mg cm^–2^, which possesses obvious advantages over previous work. The 2D layered Ni_3_(HITP)_2_ was also used for separator modification through a vacuum filtration method by Chen *et al.* [64].

Recently, Tian *et al.* prepared ultrathin Cu-based MOF (Cu_2_(CuTCPP)) nanosheets with few molecular layers for separator modification [65]. The pre-synthesized MOF nanosheets can be well dispersed in ethanol, so it is easy to construct ordered MOF films in a large area by a simple vacuum filtration method (Fig. 4d). The separator modified by Cu_2_(CuTCPP) also showed excellent flexibility. After 50 times of bending and unfolding, there was no crack or MOF falling off (Fig. 4e). The stress strain measurement also shows that the ultimate tensile stress of a MOF separator is 4.1 MPa and the Young's modulus is 0.17 GPa. By using a MOF modified separator, the Li-S battery achieved a long-term cycle of 900 cycles at 1 C with a high capacity retention of 71.1%.

In another work, MOF-based Janus conductive/insulating microporous ion-sieving membranes (MOF/G) were designed by Liu *et al.* [66]. As shown in Fig. 4f, the synthesis approach allows the MOFs to grow high aspect ratio layers in ultimate contact with the graphene surface. The cross-section HRTEM image of the MOF/G nanosheet also confirmed that it is made of MOF nanolayers and few-layer graphene flakes. Due to the novel structure, the modified PP separator showed an excellent structural flexibility and effective polysulfide adsorption. As a result, with the functional separator, long-term cycling of 1700 cycles was achieved with a capacity retention of 75.3%. At present, due to the complex synthesis methods and low yield of 2D MOF nanosheets, this strategy provides a new idea for the application of 2D MOFs in Li-S battery separator coating.

### Novel separator

Due to the mature industry of commercial polyolefin separators, the most practical, efficient and widely used modification method of polyolefin separators is adding a coating layer. However, this method will not change the essence of polyolefin separators and the modified layer will inevitably increase the areal weight and thickness of the separator, thus reducing the overall energy density of the battery. Therefore, some research began to explore the preparation of novel separators with special structure and properties for Li-S batteries [67–70]. A MOF@GO membrane was first employed as an ionic sieve for the separator of Li-S batteries by Zhou *et al.* (Fig. 4g) [71]. As shown in Fig. 4h, the GO@MOF separator is prepared by a simple vacuum filtration method. In the process of repeated filtration, HKUST-1 nanoparticles continue to grow and fill the existing space without obvious gaps between the grain boundaries. Finally, the MOF@GO composite membrane is removed from the filter membrane and directly used as the separator of the Li-S battery. The composite separator can effectively inhibit the shuttling of polysulfide to the anode without affecting the Li^+^ transport. This work provides a new idea for the application of MOFs in Li-S batteries, and also provides guidance for the application of MOF-based membranes in battery separators. However, GO may be partially reduced to conductive rGO during the cycle, resulting in the risk of short circuit. At the same time, the poor mechanical properties of composite separators would not meet the requirements of practical application. Therefore, based on this work, a novel MOF@PVDF-HFP separator for both polysulfide trapping and lithium metal anode protection was prepared by using a similar method [72]. Compared with the MOF@GO separator, the MOF@PVDF-HFP separator has better flexibility. Due to the synergistic effect of the dual function, the Li-S battery based on the composite separator exhibits excellent cycle stability; after 2000 cycles at 2 C, the capacity fading rate is only 0.015% per cycle (Fig. 4i). In order to verify the superiority of the composite separator, the performance of the pouch cell was tested (Fig. 4j). Even with an areal sulfur loading of 5.8 mg cm^–2^, the Li-S battery can still deliver a high capacity of 936 mAh g^–1^ after 200 cycles at 0.1 C, which further proves the practicability of the separator (Fig. 4k). A UiO-66-SO_3_Li-modified poly(vinylidene fluoride) (PVDF) composite separator was also prepared via the mixed-matrix membrane approach by Wang *et al.* [73].

Recently, our group also designed a ZIF-67-modified nanofiber membrane for Li-S battery separators [74]. Based on electrospinning and the following LPCVD of MOFs, a functional PAN-based separator was prepared (Fig. 4l). The PAN-based separator has good electrolyte wettability, abundant pores and high surface area, which ensure high ion conductivity and physisorption of polysulfides. It also possesses better mechanical properties and thermal stability than the PP separator. The MOF particles grown *in situ* are closely attached on the surface of the nanofibers, and these exposed particles can maximize the utilization for trapping polysulfides through chemisorption. The effect of different separators on polysulfide permeation was observed directly through permeation experiments with a double-L device (Fig. 4m). Moreover, the rGO-PAN layer can temporize the transport of the flux of Li ions to the anode surface, which can protect the Li anode and realize the long cycle life of the cell. In another work, ZIF-67- and HKUST-1-modified PMIA membranes were also employed as a functional separator for Li-S batteries [75].

More recently, a 2D MOF was also used to prepare a MOF-based novel separator. Li *et al.* reported a single atom array mimic on an ultrathin Co-MOF nanosheet-based bifunctional separator for achieving stable cycle of Li-S batteries (Fig. 4n) [76]. For the anode, because the O atoms on the surface can strongly adsorb Li^+^, the 2D Co-MOF can homogenize the Li^+^ deposition and reduce the growth of lithium dendrites; for the cathode, the Co-single atom array on the surface of the 2D MOF can effectively adsorb polysulfides and improve the utilization rate of sulfur. The excellent performance of the functional separator was also proved by the electrochemical performance test results. With the functional separator, the Li/Li symmetrical cell exhibits low polarization voltage under different current density, and the Li-S battery also shows good cycle stability.

In a word, both MOF particles and 2D MOF nanosheets can be used to modify polyolefin separators or develop novel separators for Li-S batteries. Functional separators based on MOFs can effectively alleviate the shuttle effect of polysulfides and improve the cycle stability of Li-S batteries. The key results relevant to separator coating or novel separators are summarized in Table 1. Considering the overall energy density of the battery, the loading and thickness of coating layer or novel separator must be considered. Most of the work on MOF-coated separators has paid attention to these two key parameters, while the work on novel separators often ignores the areal loadings. As we know, the ratio of electrolyte to sulfur (E/S ratio) has an important influence on the energy density of the battery. If a suitable E/S ratio is not used by MOF-based separators, the gain will not cover the loss. However, as shown in Table 1, information on the E/S ratio is often missing from reported works. Meanwhile, although MOF-based separators have improved on the cycle stability of Li-S batteries at the laboratory level, considering the prospect of commercial application, it is necessary to test the performance of Li-S batteries under extremely high sulfur loading conditions. However, there are few related works testing the performance with a high sulfur loading cathode, let alone the packaging of pouch cells. The above key parameters and necessary tests are important bases for the subsequent evaluation of MOF-based separators.

**Table 1.** Detailed information of Li-S batteries fabricated with MOF-modified polyolefin separators or novel separators.

								Cycling performance					
Samples	MOFs	Loading (mg cm^–2^)	Thickness (μm)	Cathode	Areal S loading (mg cm^–2^)	Maximum capacity (mAh g^–1^)	Rate capacity (mAh g^–1^)	Current density (C rate)	Cycle number	Fading rate (%)	Electrolyte/sulfur ratio (μL mg^–1^)	High loading test (mg cm^–2^)	Ref.
**MOF practices modified separator**													
Y-FTZB/PP	Y-FTZB	1.20	10	Super P/S	1.0	1101 (0.25 C)	N/A	0.25	300	0.165	N/A	N/A	[52]
HKUST-1/PP	HKUST-1	1.20	10	Super P/S	1.0	1032 (0.25 C)	N/A	0.25	300	0.270	N/A	N/A	[52]
ZIF-7/PP	ZIF-7	1.20	10	Super P/S	1.0	1025 (0.25 C)	N/A	0.25	300	0.186	N/A	N/A	[52]
ZIF-8/PP	ZIF-8	1.20	10	Super P/S	1.0	989 (0.25 C)	N/A	0.25	300	0.198	N/A	N/A	[52]
Ms-9.0-NSP/PP	HKUST-1	0.12	N/A	Ketjen black/S	2.4	1316 (0.5 C)	963 (2 C)	1.0	1000	0.022	N/A	5.1	[53]
								2.0	1000	0.026			
MIL-125(Ti)/PP	MIL-125(Ti)	N/A	20	Ketjen black/S	2.0	1218 (0.2 C)	592 (2 C)	0.2	200	0.202	18	N/A	[54]
UIO-66/PP	UIO-66	N/A	20	Super P/S	1.5	1120 (0.1 C)	461 (2 C)	0.5	500	0.086	13	N/A	[55]
MOF/CNT	ZIF-8	0.90	15	AB/sulfur	1.2	1655 (0.1 C)	583 (2 C)	0.2	100	0.367	40	N/A	[56]
Ce-MOF/CNT/PP	Ce-MOF	0.40	8	Ketjen black/S	2.5	1141 (0.2 C)	663 (4 C)	1.0	800	0.022	16	6.0	[61]
**2D MOF-modified separator**													
Ni_3_(HITP)_2_/PP	Ni_3_(HITP)_2_	0.07	0.34	Carbon black/S	3.5	1186 (0.2 C)	589 (5 C)	1.0	500	0.032	10	8.0	[63]
Ni_3_(HITP)_2-_/PP	Ni_3_(HITP)_2_	0.33	8	Super P/S	N/A	1220 (0.1 C)	800 (2 C)	0.5	300	0.117	N/A	N/A	[64]
Cu_2_(CuTCPP)/PP	Cu_2_(CuTCPP)	0.10	25	Carbon black/S	2.0	953 (0.2 C)	437 (5 C)	1.0	900	0.032	10	10.0	[65]
HAR-MOF/G/PP	ZIF-67	0.39	3	Super P/S	3.0−3.5	1400 (0.2 C)	400 (3.2 C)	2.0	1700	0.015	5	8.0	[66]
**Novel separator**													
MOF@GO	HKUST-1	N/A	15	CMK-3/S	0.3	1072 (0.2 C)	488 (3 C)	0.5	500	0.058	N/A	N/A	[71]
								1.0	1500	0.019			
MOF@PVDF-HFP	HKUST-1	N/A	28	RGO/S	1.0–1.5	1322 (0.1 C)	633 (3 C)	0.5	600	0.055	30	5.8	[72]
								2.0	2000	0.015			
MMMS	UiO-66/SO_3_Li	N/A	300	CMK-3/S	2.0	1069 (0.1 C)	552 (5.0 C)	0.5	500	0.056	20	N/A	[73]
MOF-PAN/rGO-PAN	ZIF-67	0.50	135	RGO/S	2.6	1302 (0.5 C)	485 (5.0 C)	5.0	600	0.032	20	7.7	[74]
F-ZIF-67-PMIA	ZIF-67	N/A	40	Carbon nanofiber/S	1.2	1268 (0.5 C)	830 (2.0 C)	0.5	500	0.091	17	N/A	[75]
F-Cu-BTC-PMIA	Cu-BTC	N/A	40	Carbon nanofiber/S	1.2	1272 (0.5 C)	881 (2.0 C)	0.5	500	0.081	17	N/A	[75]
B/2D MOF-Co	Co-BDC	0.40	25	Carbon/S	1.5	1138 (0.1 C)	478 (5.0 C)	1.0	600	0.070	20	7.8	[76]

## MOF-BASED ELECTROLYTE

Different from the traditional Li^+^ battery and other lithium metal battery systems, the electrolyte of Li-S batteries has a much more important impact on performance due to the existence of soluble polysulfides. At present, ether-based electrolytes with LiTFSI as the solute are mainly used in Li-S batteries [77,78]. However, this kind of electrolyte has high solubility of polysulfides and cannot effectively realize the uniform deposition of Li^+^. As a result, with conventional ether-based electrolytes, the problems of serious shuttle effect, the formation of unstable SEI film and the growth of lithium dendrites still exist in Li-S batteries. Therefore, the problems of Li-S battery electrolytes have been widely concerning and a lot of work on electrolyte modification [79], new electrolyte systems [80] and SSEs [81,82] for Li-S batteries has emerged. In theory, MOFs with ordered porous channels can accommodate a variety of liquid species and achieve ideal molecular/ion sieving/transport effects. Therefore, if a MOF is used as the electrolyte additive, orderly ion transport may be achieved, thus achieving uniform lithium deposition. Based on the same principle, MOFs have also been widely used as the additive for ether-based electrolytes [83,84] and the filler of SSEs to protect lithium metal anodes [85–90]. For Li-S batteries, the SSEs should be able to effectively inhibit the shuttle effect of polysulfides in addition to protecting the lithium metal anode. In this part, the application of MOFs in the modification of ether-based electrolytes and SSEs of Li-S batteries is summarized.

### Electrolyte additives

Compared with developing a new electrolyte system, adding additives to conventional electrolyte systems to improve the performance and meet the needs of practical use is a much simpler and more effective strategy in electrolyte research. Therefore, in the beginning, MOFs were used as an additive to improve the performance of ether-based electrolytes. Taking the size of TFSI^–^ into consideration, the conventional Cu-based MOF (HKUST-1) was employed as the potential host by Bai *et al.* [91]. Firstly, the barriers of TFSI^–^ anions migrating along different paths in the MOF skeleton were obtained by DFT calculation. It is concluded that TFSI^–^ anions must continuously adjust the direction when migrating in the MOF. Furthermore, the diffusion kinetics of lithium ion and TFSI^–^ anions in MOF-modified electrolytes was verified by molecular dynamics simulations. Different from the rapid diffusion of Li^+^ and TFSI^–^ in the conventional electrolyte, there are a lot of highly mobile Li^+^ and almost ‘caged’ TFSI^–^ in the modified electrolyte, which will accelerate and homogenize the deposition of Li^+^ and reduce the formation of lithium dendrites. The experimental results are also consistent with the theoretical calculation. This work proved the potential role of MOFs in realizing the tunable ion transportation of electrolytes, which would be of great significance for the electrolyte modification of Li-S batteries. Recently, based on similar principles, MIL-100 (AL) has also been used as a particulate anion sorbent to modify ether-based electrolytes for stable lithium metal batteries by Shen *et al.* [84].

### Solid-state electrolyte

Although great progress has been made in Li-S batteries based on liquid electrolytes, considering the problems of continuous consumption and thermal instabilities of liquid electrolytes in practical application, developing SSEs instead of liquid electrolytes to construct solid-state Li-S batteries is considered one of the most promising strategies. In the past few years, different kinds of solid electrolytes, including ceramic-based solid electrolytes and polymer solid electrolytes, have been used to develop solid-state Li-S batteries [92–94]. However, in addition to good ionic conductivity and excellent chemical and thermal stability, the solid electrolyte of an Li-S battery must be able to effectively inhibit the shuttle effect and protect the lithium metal anode. In order to fully meet these requirements, some fillers must be added to conventional SSEs; MOF is one of them. The MIL-53(Al)-modified polymer solid electrolyte was first prepared by Zhang *et al.* for inhibiting the shuttle effect [95]. With a PANI@C/S-280 cathode, the battery realized stable cycle performance at a high temperature of 80°C. After that, Zhou *et al.* also studied another kind of Al-based MOF-modified PEO-based polymer solid electrolyte for Li-S batteries [96]. However, the electrochemical performance of solid-state batteries at room temperature has not been studied yet.

**Figure 5.**
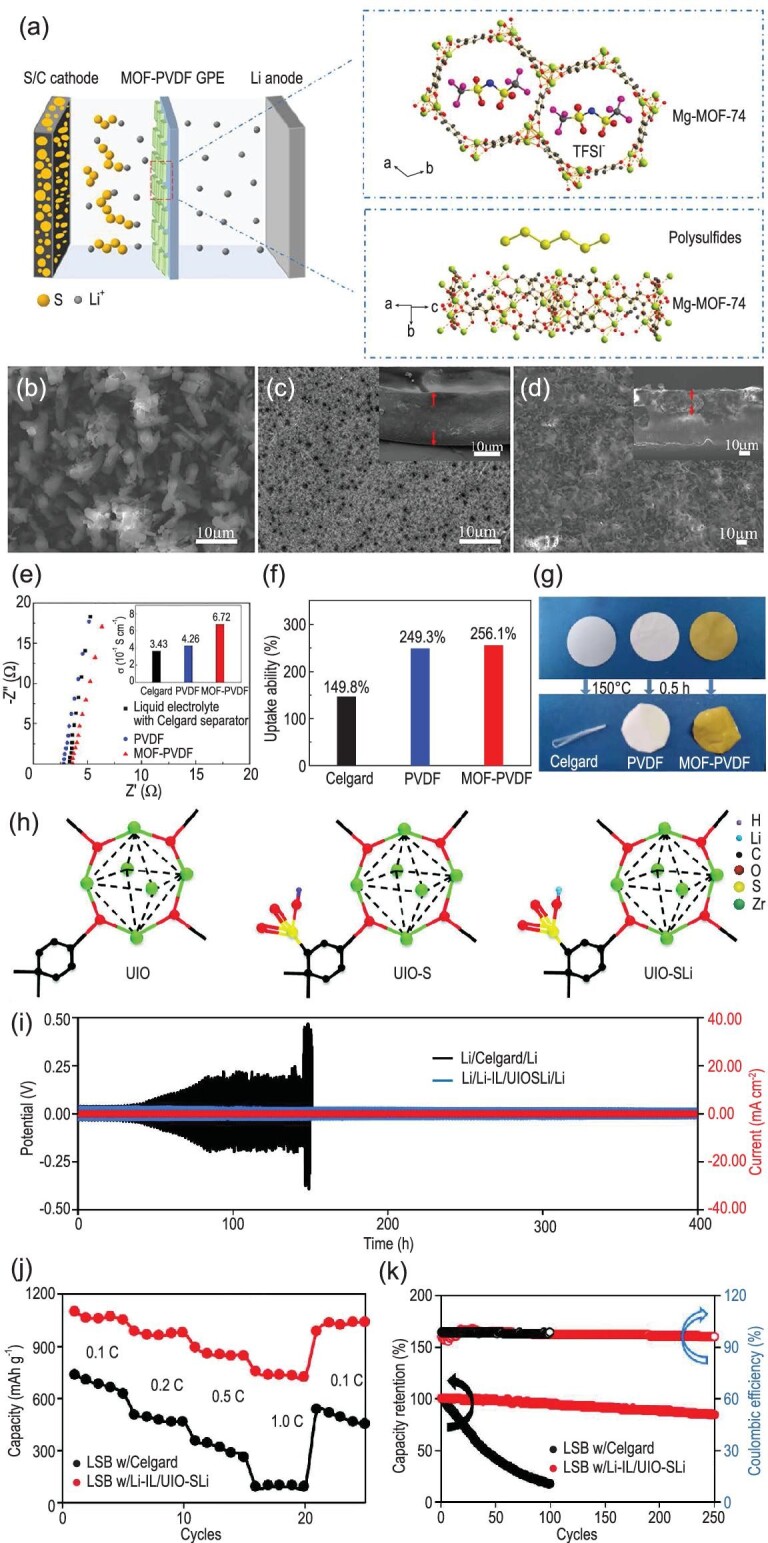
(a) Schematic illustration of MOF-PVDF GPE for the Li-S battery. SEM images of the prepared (b) Mg-MOF-74, (c) PVDF film and (d) MOF-PVDF film. Insets in (c and d) display the corresponding cross section of the bare PVDF and MOF-PVDF films, respectively. (e) The impedance spectra of the cells with various electrolytes and the relevant ionic conductivity at room temperature. (f) The uptake capacity of all membranes and (g) the photographs of the as-prepared films after heating at 150°C for 30 min. Adapted with permission from [97]. Copyright 2019, American Chemical Society. (h) Schematic structures of different UIO-66-based materials. (i) Long-term cycling profiles of different symmetric cells. (j) Discharge capacities of different cells at various C rates. (k) Long-term cycling performance of different cells at a cycling rate of 0.2 C. Adapted with permission from [98]. Copyright 2020, Wiley-VCH.

Recently, Mg-MOF-74 was chosen as a filler for PVDF-based polymer SSEs (Fig. 5a) by Han *et al.* [97]. The rod-like MOF with various lengths is distributed uniformly in the PVDF film. Compared with pure PVDF film, a more compact and stable film is obtained after modification (Fig. 5b–d). Although the thickness of the modified film is larger than that of the unmodified one, its impedance value is only slightly higher. However, due to the introduction of the MOF, its ionic conductivity is as high as 6.72 × 10^−4^ S cm^−1^, which is significantly higher than that of the pure PVDF and PP separator (Fig. 5e). At the same time, compared with the PP separator, the PVDF-based polymer solid electrolyte possess a higher electrolyte uptake and excellent thermal stability, especially after being modified by a MOF (Fig. 5f and g). Due to the addition of MOFs, the diffusion of soluble polysulfides was effectively limited by the novel polymer SSE, thus leading to a stable cycle of the Li-S battery at room temperature. Meanwhile, the MOFs with appropriate pore size could encage almost all of the TFSI^–^ anions, so as to homogenize the deposition of Li^+^. The Li/Li symmetrical cell also shows smaller voltage overpotential and superior cycle lifespan with MOF-PVDF polymer electrolyte. The protective effect of SSEs to the lithium metal anode was also proved by the smooth surface of the lithium metal anode after cycling.

In addition to polymer-based solid electrolytes, more recently a lithium sulfonate (-SO_3_Li) group grafted UIO-66 structure was used for constructing hybrid-electrolyte Li-S batteries (Fig. 5h) [98]. Unlike the filler role of MOFs in polymer-based solid electrolytes, in this work the functionalized MOFs were directly used as the framework of the solid electrolyte to separate the cathode and anode. This structure can selectively homogenize the transport of Li^+^ but block the permeation of polysulfides. As a result, the symmetrical cell showed a stable lithium stripping/plating process (Fig. 5i). Meanwhile, with Li_2_S_6_ catholyte as the active material, the hybrid-electrolyte Li-S batteries had improved rate performance (Fig. 5j) and long-term cycling performance (Fig. 5k). This work presents a new role of MOFs in SSEs.

In brief, the application of MOFs in the electrolyte of Li-S batteries has not been well developed, and the reaction mechanisms have not been fully understood. However, judging by the current research progress, MOFs will play a very promising role in the development of Li-S battery electrolytes, especially for the development of new-type SSEs.

## CONCLUSION AND OUTLOOK

This review summarizes the application of MOFs in various parts of Li-S batteries, including the cathode host, separator and electrolyte. Through an analysis of recent advances in MOF application in Li-S batteries, the working mechanisms of MOFs in different parts of Li-S batteries have been discussed in detail. The factors affecting the performance of MOFs, including the ability to adsorb polysulfides and the role of homogeneous ion deposition, have also been analyzed. Although exciting progress has been made in the application of MOFs in Li-S batteries in the past decade, the development of MOFs in Li-S batteries, especially the commercial development, is still limited by problems, and some important mechanisms are not fully understood. Therefore, key factors and potential development directions are as follows:

- The influence of MOF pore structure on Li-S batteries should be considered from more angles. The pore size of MOFs has an important influence on the performance of MOF-based composite sulfur cathodes. The existing studies only consider the limitation of pore size to polysulfide, but rarely consider the effect of pore size on the uniformity of sulfur loading by melting method and the overall sulfur content of MOF materials. However, most MOFs cannot achieve uniform sulfur loading and high sulfur content. Therefore, these factors also need to be considered when selecting the MOF with appropriate pore size.
- Design and construct the uniform and complete MOF coating layer on the separator. Previous studies have also found that when MOFs are used for separator modification, the packing density, pore size and chemical properties of MOF coatings have a great influence on its capacity to block polysulfides. However, in order to select a MOF as an ion sieve to selectively homogenize Li^+^ but block polysulfides, it is necessary to build a continuous and complete MOF film without grain boundaries on the separator. The current methods of separator modification, including vacuum filtration and *in situ* modification, cannot meet this requirement. Therefore, new methods must be used for MOF coatings. Chemical vapor deposition, magnetron sputtering and atomic layer deposition may be promising methods because they can closely arrange the precursor on the substrate to form a dense layer, which lays the foundation for the subsequent formation of a dense film of MOFs.
- Develop a new method for large-scale preparation of MOF-based separators. Although MOF-based separators significantly improve the cycle stability of Li-S batteries, due to the intrinsic frangibility of MOF crystals it is difficult for MOF-based membranes to meet the needs of practical applications. At the same time, methods of constructing MOF-based membranes are generally complex, and it is difficult to realize large-scale production. Therefore, it is necessary to further study suitable methods of large-scale production of robust and flexible MOF-based separators.
- MOF-based electrolytes will be a potential development direction. At present, only a few MOFs have been selected for the modification of ether-based electrolytes or the development of solid electrolytes for Li-S batteries, and the working mechanism is not fully understood. Therefore, it is necessary to select more kinds of MOFs for the development of Li-S battery electrolytes. Meanwhile, combining theoretical calculation with experimentation to explore new working mechanisms of MOFs in electrolytes will promote the better utilization of MOFs in Li-S batteries.
- Choose more appropriate strategies to apply MOFs to lithium metal anode protection for Li-S batteries. MOFs have been used as artificial SEI films for lithium metal anode protection [99]. In the opinion of the authors, due to the existence of polysulfides, if MOFs are used as artificial SEI films for the lithium metal anodes of Li-S batteries, although the side reaction between the polysulfides penetrating through the separator and lithium metal anode will be reduced, they will be absorbed by the polar MOFs, resulting in sulfur loss. Therefore, MOFs with adsorption effect on polysulfides are not suitable for the construction of artificial SEI films for the lithium metal anodes of Li-S battery systems. As mentioned above, using MOFs as separator coating layers or electrolyte additives will be effective strategies in protecting the lithium metal anodes of Li-S batteries.
- Low-cost and large-scale fabrication methods of MOF materials should be developed to promote their industrialization. The industrial production of MOF materials will greatly improve their application prospects in the field of commercial Li-S batteries. The production cost of MOF materials mainly comes from the raw materials and production process, while the cost and yield further affect the industrialization of MOF materials. Therefore, raw materials that are abundant in the earth should be selected in the preparation of MOF materials. At the same time, synthesis methods should be convenient, with high yield and fewer by-products. At present, the synthesis of MOF materials mainly adopts the liquid-phase method. Although the operation is relatively simple, the long production process and low efficiency greatly increase the cost. New synthesis strategies with a shorter production process and higher yield, such as the solid-solid and solid-gas methods, will promote the industrial production of MOF materials.
- Develop commercial Li-S batteries with high energy density based on MOF materials. As we know, MOF-based separators can effectively inhibit the shuttle of polysulfide and achieve uniform deposition of lithium ion, which can well inhibit the consumption of electrode materials. It is very promising to achieve stable cycling under the condition of limited N/P ratio and high sulfur load mass. At the same time, the mass of separator and electrolyte cannot be ignored when calculating the energy density of the whole cell. MOFs can hold the electrolyte well due to the appropriate pore structure on the surface, and have good wettability for the electrolyte, which can effectively improve the utilization rate of the electrolyte and reduce the electrolyte/sulfur ratio. Significantly, when MOFs are applied to modify or develop novel separators, the thickness and quality of the coating layer or separator must be reduced as much as possible, so as not to affect the overall energy density of the battery.

Despite the existing challenges, the application of MOFs in Li-S batteries has been rapidly developed, which also points out a better direction for the development of novel MOF-based materials for Li-S batteries in the future. Choosing low-cost MOF materials suitable for large-scale industrial production and developing commercial Li-S batteries with high energy density and good cycle stability will be the most important research direction. The systematic summary and profound prospects highlighted in this review will provide an important basis for the precise construction of new MOF-based cathodes, large-scale preparation of MOF-based functional separators and rational design of novel MOF-based electrolytes for Li-S batteries in the future.

## Supplementary Material

nwab055_Supplemental_File
