# Supplementary material for: Metal-organic frameworks enable broad strategies for lithium-sulfur batteries
Source: Natl Sci Rev. 2021 Apr 15;8(12):nwab055. doi: 10.1093/nsr/nwab055 (PMC8692935; doi:10.1093/nsr/nwab055)
Supplement: nwab055_Supplemental_File [file nwab055_supplemental_file.docx]

**Teaser text**

This review summarizes the recent advances of metal-organic frameworks in lithium sulfur batteries, providing feasible solutions to overcome the challenges and the guidance for the further development.
